# Multimodal imaging of apical septal rupture after anterior myocardial infarction: a case report

**DOI:** 10.1093/ehjcr/ytaf534

**Published:** 2025-10-15

**Authors:** Kazunori Omote, Tamaki Kudo, Makishi Maeda, Kenji Yamazaki, Naohiro Funayama

**Affiliations:** Department of Cardiology, Hokkaido Cardiovascular Hospital, Minami-27 Nishi-13, Chuo-ku, Sapporo 0640927, Japan; Department of Radiology, Hokkaido Cardiovascular Hospital, Sapporo 0640927, Japan; Department of Anaesthesiology, Hokkaido Cardiovascular Hospital, Sapporo 0640927, Japan; Department of Cardiothoracic Surgery, Hokkaido Cardiovascular Hospital, Sapporo 0640927, Japan; Department of Cardiology, Hokkaido Cardiovascular Hospital, Minami-27 Nishi-13, Chuo-ku, Sapporo 0640927, Japan

**Keywords:** Computed tomography, Echocardiography, Four-dimensional imaging, Ventricular septal rupture

## Case description

**Figure 1:**
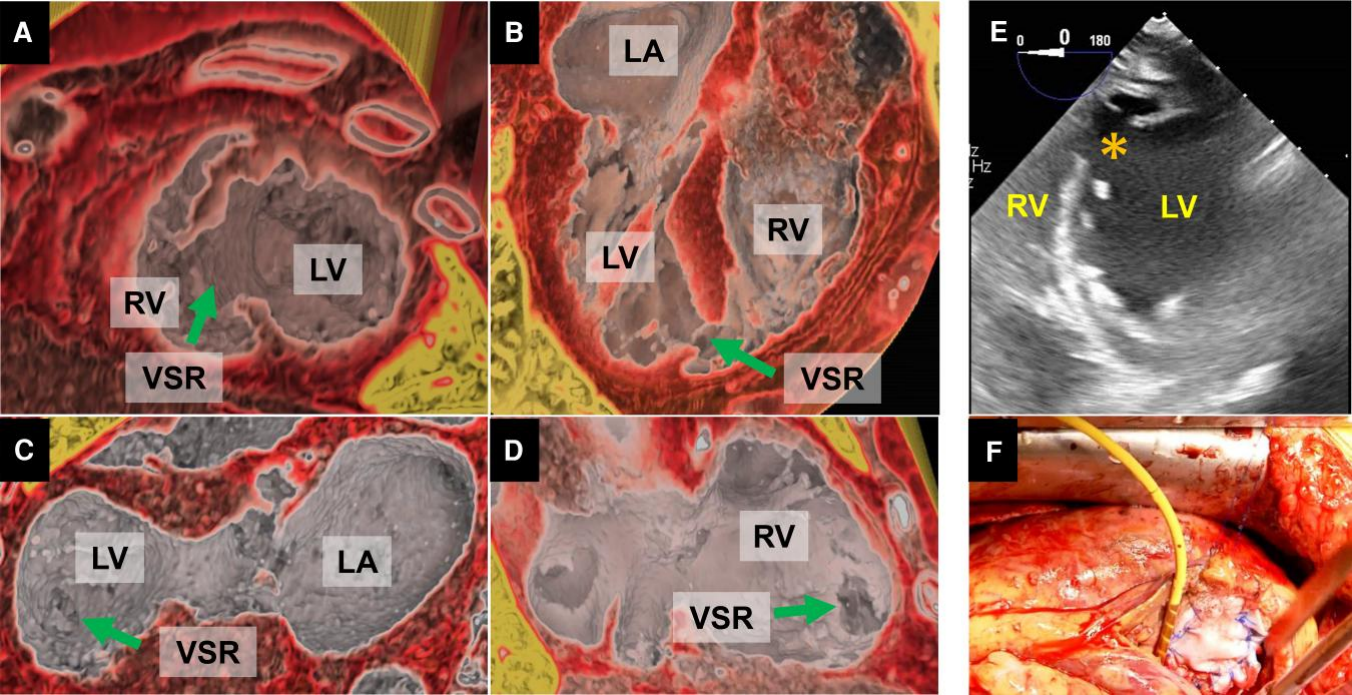
Three-dimensional computed tomography reconstructions showing cross-sectional views (*A*, *B*) and en face view (*C*, *D*) of the infarcted, thinned apical septum and the rupture tract. Intraoperative transoesophageal echocardiography visualizing the apical ventricular septal rupture (*E*). Intraoperative findings of apical ventricular septal rupture and patch repair (*F*). LV, left ventricle; LA, left atrium; RV, right ventricle; VSR; ventricular septal rupture. Asterisk and arrows indicate VSR.

A 77-year-old woman presented with cardiogenic shock following acute anterior myocardial infarction due to total occlusion of the proximal left anterior descending artery. Transthoracic echocardiography suggested an apical ventricular septal rupture (VSR) with left-to-right shunting, but the precise morphology was unclear because of poor acoustic windows. Three-dimensional (3D) echocardiography confirmed the septal defect and enabled en face visualization, although spatial resolution was limited. Colour Doppler further demonstrated systolic flow across the rupture (see Supplementary material online, *Video S1*). Contrast-enhanced 3D volume-rendered computed tomography (CT) vividly delineated the rupture tract and surrounding myocardium, providing comprehensive spatial information essential for surgical planning. The 3D reconstruction allowed intuitive appreciation of the rupture morphology and its spatial relationship to the ventricular apex, thereby facilitating operative strategy (*Figure 1A–D*). Intraoperative transoesophageal echocardiography and direct surgical inspection confirmed the apical rupture, which was successfully repaired with a patch closure (*Figure 1E and F*).

Post-infarction VSR remains one of the most devastating mechanical complications of acute myocardial infarction, with mortality exceeding 40% despite surgical intervention.^1^ Although the incidence has decreased in the reperfusion era, it continues to occur even after timely primary percutaneous coronary intervention.^2^ VSR exemplifies the spectrum of structural heart disease, where precise anatomical understanding is pivotal for management. Echocardiography remains indispensable for rapid bedside diagnosis, but image quality is often restricted in hemodynamically unstable patients. In such cases, 3D volume-rendered CT offers unique spatial resolution that complements echocardiography, enables detailed anatomical characterization, and directly informs surgical decision-making.^3^

This case highlights the incremental value of 3D volume-rendered CT in the multimodality imaging armamentarium for mechanical complications of myocardial infarction, demonstrating how advanced imaging can improve procedural planning and patient outcomes.

## Supplementary Material

ytaf534_Supplementary_Data

## Data Availability

No new data was generated or analysed in support of this research.

## Notes

**Consent:** Written informed consent for publication was obtained from the patient.

**Funding**: None declared.
